# Pregnancy After Panniculectomy Postbariatric Surgery: A Case Report

**DOI:** 10.7759/cureus.68138

**Published:** 2024-08-29

**Authors:** William West, Valerie C Nemov, Nicole K Le, Kristen Whalen, Deniz Dayicioglu, Bri Anne McKeon

**Affiliations:** 1 Department of Medicine, University of South Florida Health Morsani College of Medicine, Tampa, USA; 2 Department of Plastic Surgery, University of South Florida Health Morsani College of Medicine, Tampa, USA; 3 Department of Obstetrics and Gynecology, University of South Florida Health Morsani College of Medicine, Tampa, USA

**Keywords:** preterm birth, massive weight loss, abdominoplasty, abdominal body contouring, bariatric surgery, panniculectomy, pregnancy outcomes

## Abstract

Panniculectomy and abdominoplasty are two forms of abdominal body contouring commonly performed. There are literature reports of healthcare providers citing abdominoplasty as a contraindication to future pregnancy due to potential risks to the patient and fetus. Panniculectomy, outside of the reduced risks from the lack of rectus muscle plication, would have similar effects on the patient and fetus in future pregnancies. Yet, abdominal contouring surgery is being performed in women of childbearing age with increasing frequency, meriting further research to explore the true safety profile of pregnancy after body contouring surgeries. Here, we present a case report of a woman who underwent two full-term vaginal deliveries after undergoing a panniculectomy due to massive weight loss from prior gastric bypass. While she had high utilization of healthcare services throughout her pregnancies, she experienced no significant adverse pregnancy outcomes. Our report is consistent with current literature, suggesting that prior panniculectomy should not be a contraindication to pregnancy.

## Introduction

Abdominal contouring surgeries, such as panniculectomy and abdominoplasty, are regularly performed plastic surgery procedures that greatly enhance patients' quality of life. Following massive weight loss or pregnancy, these procedures may improve problematic lower abdominal deformities such as excess skin and tissue laxity, which can lead to discomfort, rash, infection, and distressing esthetic appearance. As the rates of obesity and subsequent bariatric surgery and weight loss continue to increase, so does the rate of abdominal body contouring performed in women of childbearing age [1,2].

Prior research on pregnancy after abdominal body contouring has focused on abdominoplasty. There are literature reports of women seeking abdominoplasty being advised to do so after they have completed childbearing [2]. Concerns for pregnancy after abdominoplasty are two-fold: pregnancy may disrupt the esthetic outcome of abdominoplasty, and prior abdominoplasty may predispose both the patient and fetus to complications. Specifically, a surgically tightened abdominal wall may present an undesirable restriction to the abdominal expansion necessary to accommodate an enlarging gravid uterus [1,2].

Panniculectomy is a less extensive surgery than abdominoplasty. In contrast to abdominoplasty, which is performed for cosmetic purposes, panniculectomy is performed for functional reasons to remove an overlying abdominal panniculus; it does not involve surgical tightening of the abdominal musculature [3]. A panniculectomy is more likely to be the surgery of choice following bariatric surgery due to insurance coverage and lower surgical risk with skin resection alone [4]. Thus, patients who conceive after panniculectomy are more likely to have undergone bariatric surgery. A history of bariatric surgery may further increase the risk of pregnancy secondary to resulting nutritional deficiencies [5].

The literature on pregnancy following abdominoplasty is sparse, and the literature on pregnancy following panniculectomy after bariatric surgery is nonexistent. There are no formal guidelines on how obstetricians and plastic surgeons should advise their patients on pregnancy after any type of abdominal body contouring. Here, we present a case report of a woman with two full-term pregnancies following panniculectomy after Roux-en-Y gastric bypass. We add to the currently limited literature that pregnancy after abdominal contouring surgery, including panniculectomy, appears safe. The patient provided consent for her case to be shared.

## Case presentation

A 34-year-old G2P2002 Caucasian female presented to the plastic surgery department with concerns about abdominal tissue laxity that interfered with her activities of daily living, including difficulty exercising, recurrent rashes, back pain, and issues finding clothing. She had undergone a Roux-en-Y gastric bypass surgery three years prior and subsequently lost 160 pounds (from a maximum weight of 365 pounds). Her medical history was significant for hypertension, hyperlipidemia, and obstructive sleep apnea, which all resolved following bariatric surgery. Additionally, she had various nutritional deficiencies secondary to surgery, including iron deficiency anemia. She also had a history of a right jugular deep vein thrombosis related to a port placed for her bariatric surgery, which resolved with short-term warfarin treatment.

**Figure 1:**
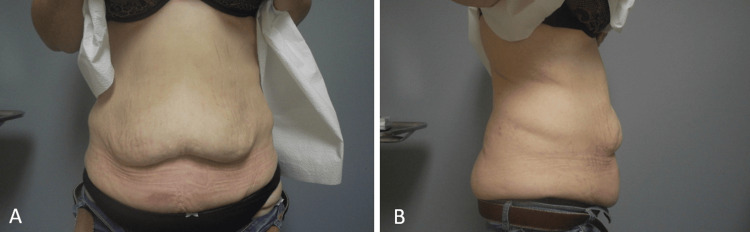
Redundant abdominal tissue prior to panniculectomy. The patient underwent a Roux-en-Y gastric bypass in 2011. During the initial plastic surgery consultation one month before panniculectomy, frontal (A) and right lateral (B) photographs of the patient were taken, demonstrating redundant tissue

The decision was made to proceed with panniculectomy to address her abdominal tissue laxity and redundant skin (Figure 1). The tissue removed weighed 2,060 g. The patient did not undergo rectus plication or tightening of abdominal musculature. Her postoperative course was complicated by significant anemia, necessitating a blood transfusion. Additionally, she presented to the emergency department four days after discharge with dizziness and again three days later with complaints of dizziness and right calf pain. There was no indication of further transfusion or evidence of deep vein thrombosis.

Approximately one year and eleven months later, the 36-year-old patient became pregnant. At the time of her initial prenatal appointment, the patient weighed 245 pounds, and her BMI was 35.1 kg/m^2^. Her first two pregnancies before her panniculectomy were complicated by preeclampsia. During this pregnancy, she presented to the hospital a total of seven times, which included one hospital admission (at six weeks zero days) and six emergency room/obstetric triage visits that did not result in admission. Her presentations were for abdominal pain/cramping, dizziness, headache, and iron deficiency anemia. One evaluation (at 22 weeks 4 days) was for vomiting, postulated to be secondary to a reduced ability for gastric distention following her surgeries. Her subsequent presentation (at 29 weeks 0 days) was for abdominal cramping, determined to be related to her recurrent history of dehydration following her gastric bypass. Her last two evaluations were for headaches and blurry vision; her blood pressure remained within normal ranges, and she was not diagnosed with a hypertensive disorder of pregnancy. At 39 weeks of gestation, the patient went into spontaneous labor resulting in a spontaneous vaginal delivery of a healthy male infant after oxytocin augmentation. Her postpartum course was unremarkable, and she was discharged on postpartum day 2.

She presented again at age 37, this time approximately three years and four months following her panniculectomy with a second pregnancy. At her initial prenatal appointment for this pregnancy, she weighed 275 pounds, and her BMI was 36.7 kg/m^2^. She was noted to have chronic hypertension, but her pregnancy was otherwise uncomplicated. She continued to utilize hospital services highly throughout this pregnancy, with five emergency department or obstetric triage evaluations. However, the majority of her presentations were due to pregnancy-related pelvic pain, pressure, and contractions, and she did not require hospital admission. She underwent induction of labor at 39 weeks of gestation due to chronic hypertension resulting in a spontaneous vaginal delivery of a healthy female infant. There were no complications, and she was discharged on postpartum day 2. The patient presented to the obstetrics and gynecology department four weeks postpartum with a posterior vaginal wall prolapse/rectocele.

Regarding her history of gastric bypass and obesity, the patient did not have a follow-up with the bariatric team between August 2013 and March 2020, when she reestablished with bariatrics for assistance with weight control two years after her last delivery. She weighed 285 pounds then and was initiated on a food management program and phentermine. Topiramate was added at a future appointment. Throughout this time, she experienced depression related to her weight, which was treated with sertraline. She underwent endoscopy due to symptoms of rapid pouch emptying, which established an anatomical deterioration of the gastrojejunal anastomosis. In February 2021, the patient underwent an endoscopic revision of her gastric bypass, subsequently lost 24 pounds (to 254 pounds), and was lost to bariatric follow-up in late 2021. Most recently, six and a half years after her second delivery after panniculectomy, the patient weighed 312 pounds.

## Discussion

This study presents the case of a woman who had two successful pregnancies after panniculectomy following a Roux-en-Y gastric bypass. This patient's risk of pregnancy was higher than that of the average pregnancy regardless of her prior panniculectomy due to her history of gastric bypass, preeclampsia, chronic hypertension, and advanced maternal age. However, she had a relatively uncomplicated course and experienced no adverse pregnancy outcomes (fetal growth restriction, preterm birth, or worsening hypertension of pregnancy) with either of her pregnancies. This case supports the existing literature that says abdominal body contouring should not be a contraindication of pregnancy.

The patient did not experience any adverse pregnancy outcomes due to her prior body contouring surgery and did not require a cesarean section. Hospitalizations throughout her first pregnancy after panniculectomy were largely for gastrointestinal symptoms and nutritional deficits related to her gastric bypass surgery. Both are known phenomena in pregnant patients after bariatric surgery and should be part of patient counseling in patients after bariatric surgery who are considering pregnancy. Postpartum, the patient had regained much of the weight that she lost following gastric bypass. Patients who undergo body contouring after bariatric surgery are known to have improved long-term weight control compared with patients who do not undergo body contouring [6]. It is possible that this patient's pregnancies contributed to her poor long-term weight control despite panniculectomy. However, previous research has indicated that pregnancy after bariatric surgery does not impact long-term weight outcomes [7,8]. The potential effect on long-term weight is another important consideration in patient counseling for patients with prior bariatric surgery considering pregnancy.

Previous literature on pregnancy following abdominal contouring focuses on abdominoplasty. Multiple literature sources assert that patients should only undergo abdominoplasty when they no longer plan to become pregnant. This conclusion is based on the concern that pregnancy after abdominoplasty may lead to adverse outcomes for the patient or fetus or endanger the aesthetic outcome of the abdominoplasty [9,10]. A published abstract identified 99 women who underwent abdominoplasty and became pregnant [11]. These women had higher rates of cesarean deliveries than the hospital and national average. A social media survey of 32 women who became pregnant after abdominoplasty revealed that 15.6% of these women experienced late preterm births, 50% reported compromised esthetic outcomes, and 6.2% underwent abdominoplasty revision after pregnancy. Additionally, this survey found that 43.8% of women would not recommend abdominoplasty before pregnancy [12].

The importance of delineating the true effects of prior abdominal body contouring on pregnancy is highlighted by the report of a woman with a history of abdominoplasty who terminated her pregnancy as advised by her doctor. Years later, the same patient was advised by a different doctor to continue another pregnancy, and she delivered uneventfully with an elective cesarean section [13]. Despite the anecdotal belief that abdominoplasty is a contraindication to pregnancy, multiple reports have detailed cases of patients who underwent successful pregnancy and delivery after abdominoplasty [10,14-17]. A national registry-based study in Finland identified 92 women with lower body contouring surgery before pregnancy [1]. They reported that while women in their study who gave birth following lower body contouring surgery were more likely to undergo cesarean section and preterm delivery, these differences were due to differing demographics between the two study groups. A recent systematic review concluded that abdominoplasty should not be a contraindication to pregnancy [2].

In comparison to abdominoplasty, there are no reports of pregnancy outcomes following panniculectomy specifically. Furthermore, only two studies on pregnancy after abdominoplasty included patients with bariatric surgery [1,2]. It is known that patients undergoing functional panniculectomy are more likely to have comorbidities and postoperative wound infection, dehiscence, sepsis, bleeding, readmission, and reoperation than patients undergoing cosmetic abdominoplasty [18]. Panniculectomy is also a functional procedure more likely to be performed on patients who underwent bariatric surgery [4]. Pregnant patients who previously had bariatric surgery are also at increased risk for nutritional deficiencies, gastrointestinal problems, and cesarean delivery [5]. Therefore, while panniculectomy does not include abdominal wall tightening as abdominoplasty does, the existing comorbidities of this patient population before pregnancy present a potential pregnancy risk. Plastic surgeons, obstetricians, and gynecologists at our institution do not advise patients against pregnancy after panniculectomy or abdominoplasty. It is important to counsel patients on the potential effects of pregnancy on their esthetic results and options for potential revision if desired. Additionally, patients should be informed of the potential increased risk for preterm labor and higher rates of cesarean sections found in some studies on pregnancy after body contouring surgery. Finally, it is of paramount importance to include contraceptive counseling in the perioperative period if further pregnancy is not desired.

This study had several limitations. The only pictures taken of the patient were before panniculectomy. Lacking images after panniculectomy, as well as before and after both pregnancies, makes it impossible to evaluate esthetic outcomes following pregnancy. Additionally, this is a report of only one patient. To draw definitive conclusions about pregnancy outcomes following panniculectomy after bariatric surgery, a prospective cohort study should be conducted.

## Conclusions

In this case, a patient underwent two successful pregnancies after a panniculectomy postgastric bypass. This case further proves that abdominal body contouring should not be a contraindication to pregnancy. It is the first case reported in the literature of a patient who underwent panniculectomy due to massive weight loss after bariatric surgery and subsequently became pregnant. We hope that this report will encourage future studies with a larger cohort of patients that will provide more definitive conclusions about the safety of pregnancy following panniculectomy after bariatric surgery.
